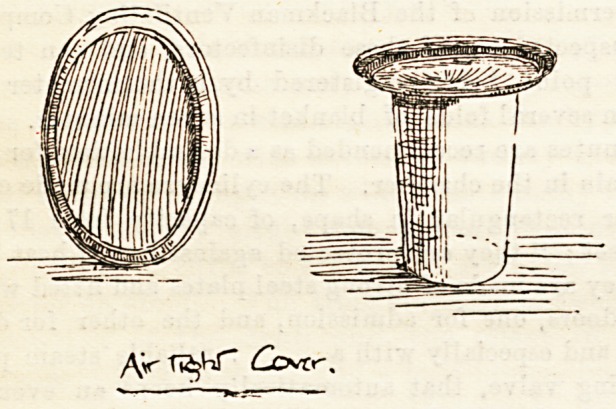# Air-Tight Cover

**Published:** 1895-06-01

**Authors:** 


### AIR-TIGHT COVER FOR SICK-ROOM USE.

Messrs. Day and Co., engineers, of Weston-super-Mare, have brought out and patented a very useful little article adapted for many purposes, and especially convenient in the sick-room, in the Air-Tight Cover, of which we give an illustration below. We do not remember to have seen the idea carried out before, though in itself it is sufficiently simple. The cover consists of a piece of very fine india-rubber stretched upon a circular metal frame, and its action depends upon atmospheric pressure. In placing it over the vessel the centre is depressed by the finger, thus expelling some of the air, and removal of the pressure causing a partial vacuum, the fixture is complete, the vessel being instantly hermetically sealed, so that it can be turned upside down or lifted up by the cover itself alone. The rim of tumbler or cup should of course be smooth, and moistened. To ensure the latter the plan advised is merely to partially invert the vessel so as to allow the contents to moisten the cover. It will be found very useful in keeping milk, beef tea, &c., free from contact with the air, and any possible contamination, and it will even keep a cup of tea, &c., hot for some time. It has also another use. To break an egg into a tumbler, apply the cover, and administer a few vigorous shakes will be found as effectual as beating with a fork, and

Air Tight Cover.

a far quicker process. The covers are sold in four different sizes, 3, 4, 5½, and 7 inches in diameter, price 6d., 9d., 1s., and 1s. 3d. respectively, and can be obtained from any chemist or ironmonger, or direct from the manufacturers, Messrs. Day and Co., Paragon Road, Weston-super-Mare. The invention is a distinctly useful one. The covers are simple in make, and can be cleaned thoroughly in a moment.

## Figures and Tables

**Figure f1:**